# Gene expression of *Ceriporiopsissubvermispora *during lignocellulosic substrate degradation

**DOI:** 10.1186/1753-6561-8-S4-P170

**Published:** 2014-10-01

**Authors:** SháskyaTanyara Alves do Nascimento Barbosa, Zenon M Lima, Priscila Almeida Calixto, Ubiratan Rios Santos, Leandro Eugênio Cardamone Diniz, Jorge A López, Rodrigo Cazzaniga, Maria Lucila Hernández-Macedo

**Affiliations:** 1 Curso de Farmácia, Universidade Tiradentes, Farolândia, Aracaju, SE, Brazil; 2 Programa de Pós-Graduação em Biotecnologia Industrial, Universidade Tiradentes/Instituto de Tecnologia e Pesquisa, Aracaju, SE, Brazil; 3 Embrapa - Tabuleiros Costeiros, Aracaju, SE, Brazil; 4 Instituto de Tecnologia e Pesquisa, Aracaju, SE, Brazil

## Background

Lignin is a complex aromatic polymer whose microbial degradation is essential for carbon recycling [1]. The fungus *Ceriporiopsis subvermispora *has attracted attention for its valuable biotechnological applications, especially for pretreatment of lignocellulosic material studies in order to expose the cellulose for hydrolysis and industrial fermentation. Thus, ligninolytic enzymes of white-rot fungi remain the subject of intensive investigations for their potential applications in a wide range of industrial bioprocesses and applications such as the paper industry, textile industry, decolorization of industrial wastewater treatment, and the degradation of organopollutants [2]. The goal of the present work was to grow *C. subvermispora *in coconut fibers as substrate in order to analyze its degradation potential and evaluate the lignin peroxidase expression (*Lip1 *and *Lip2*).

## Methods

The fungus was maintained on malt agar at 4ºC. For inoculation, a fungal mycelium suspension (300 µL) obtained from 2% malt extract medium was inoculated on 3 g of coconut fibers with 10 mL of sterile distilled water and then incubated at 28ºC at 15, 30, 45, and 60 days. Gene expression was performed by real-time PCR (qPCR) during a cultivation period between 15 to 60 days.

## Results and conclusions

At the experimental interval, rapid fungus colonization on the lignocellulosic substrate was observed. The RT-PCR analysis showed that *Lip1 *gene expression started before the15th day of cultivation. On the 15th day, this gene was highly expressed and its expression decline was observed from the 45th day. Regarding *Lip2*, its highest expression was observed in the range between 30 and 45 days of cultivation, while the decrease in the *Lip2 *expression occurred from the 45th day. Comparing both genes, an abrupt decrease in the *Lip1 *and *Lip2 *expression was observed on the 60th day of culture, inferring the possibility that these genes act simultaneously during the degradation of the substrate evaluated. Overall, the results indicate the *C. subvermispora *potential to degrade coconut fibers, and the gene expression analysis may provide data to understand the fungus extracellular system used to decompose lignin [3,4]. Also, insights on the role of regulating elements in the expression of these ligninolytic enzymes are necessary for efficient biotechnological applications.
